# Cocaine-Induced Mixed Diabetic Ketoacidosis-Hyperosmolar Hyperglycemic State Mimicking Acute Cholecystitis: A Case Report

**DOI:** 10.7759/cureus.113357

**Published:** 2026-07-25

**Authors:** Blessing G Ibokette, Manikya Nagaraja, Frederick Tiesenga

**Affiliations:** 1 Medicine, All Saints University School of Medicine, Roseau, DMA; 2 Medicine, Windsor University School of Medicine, Cayon, KNA; 3 General Surgery, West Suburban Medical Center, Chicago, USA

**Keywords:** cocaine induced, diabetic ketoacidosis (dka), distributive shock, extreme hyperglycemia, hyperosmolar hyperglycemia state, ischemic hepatitis, reversible cardiomyopathy, substance-induced metabolic acidosis

## Abstract

We present the case of a 52-year-old man with a history of diabetes mellitus who presented with altered mental status following acute cocaine use. Initial evaluation revealed severe hyperglycemia (1655 mg/dL), profound metabolic acidosis, hyperkalemia, and severe hypernatremia consistent with mixed diabetic ketoacidosis (DKA) and hyperosmolar hyperglycemic state (HHS). The patient required endotracheal intubation, mechanical ventilation, continuous insulin infusion, aggressive fluid resuscitation, and electrolyte correction. His intensive care unit course was complicated by distributive shock requiring norepinephrine, phenylephrine, and vasopressin support. He subsequently developed transient cardiomyopathy with a left ventricular ejection fraction of 35% and marked transaminitis. Right upper quadrant ultrasonography and computed tomography demonstrated gallbladder sludge and borderline wall thickening, raising concern for acute cholecystitis. However, hepatobiliary scintigraphy was negative for cystic duct obstruction, and liver enzymes improved rapidly following hemodynamic stabilization. These findings supported a diagnosis of ischemic hepatitis secondary to shock and cocaine-induced vasoconstriction rather than primary biliary pathology. The patient's clinical status improved with supportive management, and he was discharged with outpatient endocrinology and cardiology follow-up. This case highlights the potential for cocaine-associated mixed DKA-HHS to present with profound metabolic derangement and multisystem organ dysfunction while mimicking acute biliary disease, emphasizing the importance of careful diagnostic evaluation to avoid unnecessary surgical intervention.

## Introduction

Diabetic ketoacidosis (DKA) and hyperosmolar hyperglycemic state (HHS) are acute metabolic complications of diabetes mellitus associated with significant morbidity and mortality [1]. Pathophysiologically, DKA results from severe insulin deficiency, leading to lipolysis, ketone production, high anion gap metabolic acidosis, and typically moderate hyperglycemia. In contrast, HHS is characterized by relative insulin deficiency, extreme hyperglycemia (often >600 mg/dL), profound dehydration, and serum osmolality exceeding 320 mOsm/kg, with minimal or absent ketoacidosis [2].

Although traditionally considered distinct entities, mixed DKA-HHS presentations occur and are associated with more severe metabolic derangements and worse outcomes [2]. Cocaine is a recognized precipitant of hyperglycemic crises due to its sympathomimetic effects, which promote insulin resistance, increased hepatic glucose production, and systemic vasoconstriction [3]. Epidemiologic studies have identified active cocaine use as an important precipitating factor for DKA and other hyperglycemic crises.

Mixed presentations of DKA and HHS are uncommon and are associated with substantially higher morbidity and mortality than isolated DKA or HHS, particularly in the setting of severe hyperosmolarity and shock [1,3]. We present a case of cocaine-associated mixed DKA-HHS with extreme hyperglycemia complicated by ischemic hepatitis and cardiomyopathy, initially mimicking acute cholecystitis.

## Case presentation

A 52-year-old man with a history of diabetes mellitus was brought to the emergency department with altered mental status. History was unobtainable on arrival due to obtundation.

Initial laboratory studies demonstrated profound metabolic derangements. The serum glucose was 1655 mg/dL, venous pH was 6.87, bicarbonate was 7 mmol/L, potassium was 6.7 mmol/L, sodium was 125 mmol/L (corrected sodium was approximately 150 mmol/L), lactate was 3.3 mmol/L, and measured serum osmolality was 361 mOsm/kg (Table 1). Urine acetone was positive, urinalysis glucose was greater than 500 mg/dL, and urine toxicology was positive for cocaine. These findings were consistent with a mixed DKA-HHS presentation [1,2]. The patient was intubated for airway protection and admitted to the intensive care unit (ICU). Management included aggressive intravenous fluid resuscitation, continuous insulin infusion, electrolyte correction, bicarbonate therapy for severe acidosis, and broad-spectrum antibiotics.

**Table 1:** Laboratory investigations during hospitalization This table summarizes key laboratory parameters from admission through clinical stabilization. Peak/worst values represent the most abnormal measurements recorded during hospitalization. Initial serum sodium was affected by severe hyperglycemia (pseudohyponatremia), with a corrected sodium concentration of approximately 150 mmol/L. AST, aspartate aminotransferase; ALT, alanine aminotransferase; BUN, blood urea nitrogen; WBC, white blood cell count; Troponin-HS, high-sensitivity troponin. Note: An em dash (—) indicates that the laboratory test was not repeated or that the result was unavailable at the corresponding time point.

Laboratory Parameter	Admission (Day 0)	Peak/Worst Value	Day 4	Day 5	Reference Range
Glucose (mg/dL)	1655	1655	206	114	70–99
Venous pH	6.87	6.87	—	—	7.30–7.43
Bicarbonate (mmol/L)	7	7	28	31	21–31
Anion Gap (mmol/L)	40	40	4	3	3.6–11
Sodium (mmol/L)	125*	152	152	149	133–144
Potassium (mmol/L)	6.7	6.7	5.2	4.2	3.5–5.1
Chloride (mmol/L)	79	120	120	115	98–109
BUN (mg/dL)	81	81	34	24	7–25
Creatinine (mg/dL)	3.78	3.78	0.94	0.65	0.6–1.3
AST (U/L)	15	2198	1365	2198	13–39
ALT (U/L)	32	929	686	929	7–52
Total Bilirubin (mg/dL)	0.4	1.6	1.6	0.7	0–1.0
WBC (×10⁹/L)	18.8	18.8	—	6.3	4.0–11.0
Troponin-HS (ng/L)	52	52	—	—	0–20
Lipase (U/L)	101	101	—	—	11–82
Serum Acetone	Positive	Positive	—	—	Negative

During the ICU course, the patient developed significant hypotension with a nadir blood pressure of 87/35 mmHg, consistent with distributive shock in the setting of severe metabolic derangement. He required initiation of vasopressor support, including norepinephrine, phenylephrine, and vasopressin, to maintain adequate mean arterial pressure. Vasopressor requirements persisted despite aggressive fluid resuscitation and correction of metabolic abnormalities, with gradual hemodynamic improvement and successful weaning of vasopressors as the patient stabilized.

**Figure 1:**
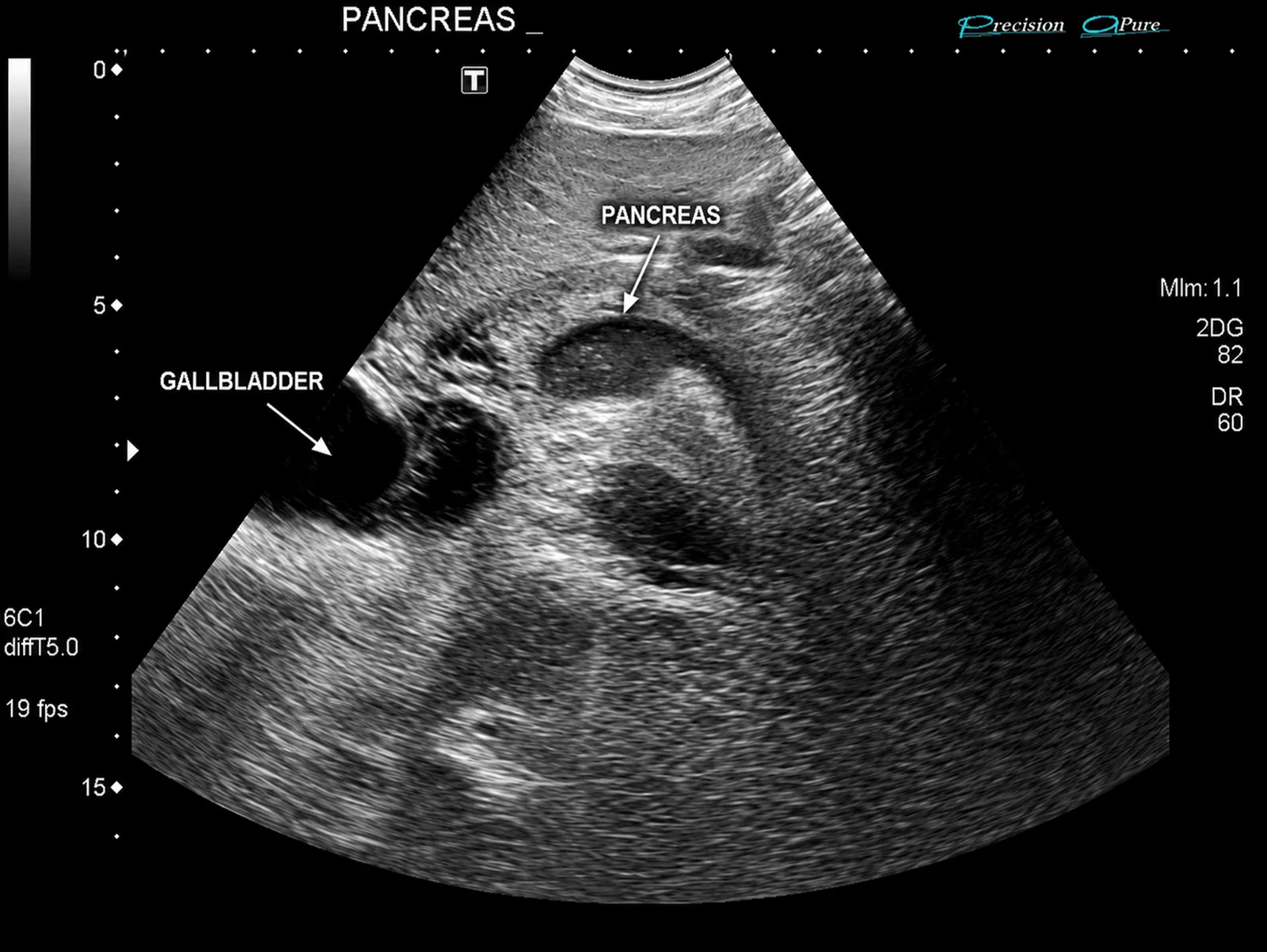
Right upper quadrant abdominal ultrasound Gallbladder sludge and borderline gallbladder wall thickening can be seen without frank sonographic evidence of acute cholecystitis, pericholecystic fluid, or biliary ductal dilatation.

**Figure 2:**
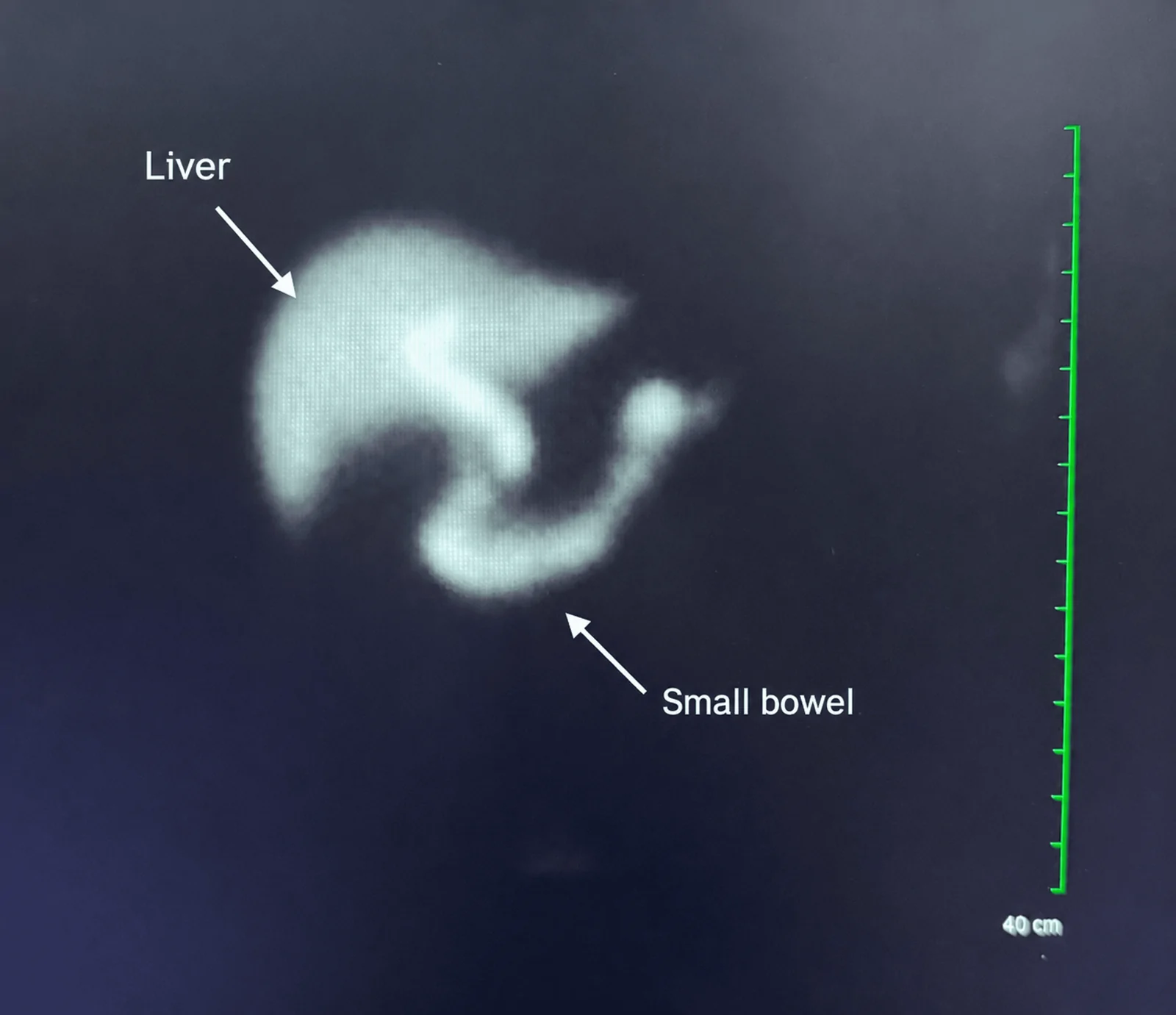
Hepatobiliary scintigraphy (HIDA scan) Prominent radiotracer uptake is seen within the liver parenchyma (indicated by the Liver arrow) and radiotracer passage into the small bowel (indicated by the Small bowel arrow). The complete HIDA examination was interpreted as showing no evidence of acute cholecystitis. HIDA, hepatobiliary iminodiacetic acid

During hospitalization, the patient developed marked transaminitis, with peak AST and ALT levels of 2,198 U/L and 929 U/L, respectively. This pattern is characteristic of ischemic hepatitis following hepatic hypoperfusion [4]. Right upper quadrant ultrasound demonstrated gallbladder sludge and borderline gallbladder wall thickening without definitive evidence of acute cholecystitis (Figure 1). Computed tomography showed similar findings, raising concern for acute cholecystitis. However, the patient lacked abdominal pain. Subsequent hepatobiliary scintigraphy was negative for acute cholecystitis (Figure 2), and the surgical service concluded that no operative intervention was indicated. Liver enzymes improved with hemodynamic stabilization, consistent with ischemic hepatitis rather than biliary pathology. 

Echocardiography revealed a reduced left ventricular ejection fraction of 35% with septal and apical hypokinesia, consistent with stress- or cocaine-associated cardiomyopathy. The patient was successfully extubated on Hospital Day 5. Despite correction of the metabolic abnormalities, vasopressor support was required for several days because of persistent hemodynamic instability and was successfully discontinued on Hospital Day 10. Renal function normalized, transaminase levels decreased, and glycemic control improved. He was discharged on Hospital Day 11. The patient was ambulating with assistance prior to discharge and was referred for outpatient endocrinology and cardiology follow-up.

## Discussion

Cocaine precipitates hyperglycemic crises through catecholamine-mediated insulin resistance, increased hepatic gluconeogenesis, and impaired peripheral glucose uptake [1]. In our patient, profound hyperglycemia (1655 mg/dL), severe acidemia (venous pH 6.87), and hyperosmolality (361 mOsm/kg) were consistent with a mixed DKA-HHS presentation. Extreme hyperglycemia exceeding 1500 mg/dL is rarely reported and is associated with severe insulin deficiency, profound dehydration, and stress hormone excess, all of which are recognized contributors to severe metabolic decompensation and were consistent with this patient's presentation [1,5].

Ischemic hepatitis typically presents with abrupt transaminase elevations following hepatic hypoperfusion [6]. Previous studies have described hepatic hypoperfusion as the underlying mechanism of ischemic hepatitis in similar clinical settings [6,7]. In our patient, persistent shock physiology together with cocaine-induced vasoconstriction was associated with hepatic hypoperfusion and marked transaminitis. The absence of cystic duct obstruction on HIDA (hepatobiliary iminodiacetic acid) scan, together with the overall clinical presentation, supported ischemic hepatitis rather than acute biliary pathology and prevented unnecessary surgical intervention.

Although the patient's lipase was mildly elevated (101 U/L), it did not exceed three times the upper limit of normal, a threshold commonly used as one of the diagnostic criteria for acute pancreatitis when interpreted in conjunction with compatible clinical findings and imaging [8]. There were also no imaging findings to support acute pancreatitis, making pancreatitis an unlikely explanation for the patient's presentation.

Cocaine toxicity and severe metabolic stress can also result in transient cardiomyopathy [9,10]. In our patient, the mildly elevated high-sensitivity troponin level, together with a reduced left ventricular ejection fraction of 35% and septal and apical hypokinesia, was consistent with stress- or cocaine-associated myocardial dysfunction [9-11]. Our patient developed profound metabolic derangements and multiorgan dysfunction; however, the marked transaminitis and imaging findings initially mimicking acute cholecystitis highlight an uncommon diagnostic challenge.

This case highlights the importance of integrating clinical context with imaging findings to avoid diagnostic anchoring and unnecessary surgical intervention. Clinicians should consider ischemic hepatitis in patients with severe hyperglycemic crises and shock who present with marked transaminitis and imaging findings suggestive of biliary disease. 

## Conclusions

Cocaine-induced mixed DKA and HHS may present with profound metabolic derangement and multisystem organ dysfunction, including distributive shock, ischemic hepatitis, and transient cardiomyopathy. This case highlights the diagnostic challenge posed by ischemic hepatic injury, which may mimic acute biliary pathology on imaging and potentially lead to unnecessary surgical intervention. Early recognition of mixed DKA-HHS, aggressive hemodynamic resuscitation, correction of metabolic abnormalities, and careful interpretation of imaging findings are essential for optimizing patient outcomes. Clinicians should maintain a high index of suspicion for ischemic injury patterns and avoid diagnostic anchoring when evaluating critically ill patients presenting with extreme hyperglycemia and concurrent cocaine use. Awareness of this uncommon presentation may facilitate earlier diagnosis and help avoid unnecessary invasive interventions in similar clinical scenarios. Patients should also receive counseling regarding cocaine cessation, with appropriate outpatient follow-up to reduce the risk of recurrent hyperglycemic crises and associated complications. 
